# Maximizing opportunities for success as an early career STEM faculty: a growth mindset approach

**DOI:** 10.1186/s12919-025-00356-y

**Published:** 2025-12-18

**Authors:** Jim O. Vigoreaux, Leslie A. Caromile

**Affiliations:** 1 Department of Biology, University of Vermont, Burlington, VT USA; 2 Department of Cell Biology and Center for Vascular Biology, University of Connecticut School of Medicine, Farmington, CT USA

**Keywords:** Academic career, Institutional culture, Career transition, STEM, Growth mindset, Faculty professional development

## Abstract

Academics are familiar with the concept of a growth mindset, a framework teachers use to help students embrace challenges, overcome obstacles, and learn from their mistakes. In this context, we argue that the principles of this paradigm are equally applicable in promoting the career advancement of early STEM academics, as well as those in non-STEM fields where successful career progression requires demonstrating contributions to scholarship, teaching, and service, often involving collaborative work. The early years of a faculty career offer numerous opportunities for growth, but these may be missed or seen as obstacles if approached with a fixed mindset. This is especially true for individuals with intersectional identities who may lack role models or feel out of place in an unsupportive academic environment. We argue that a well-planned, purposeful, and balanced approach to workload responsibilities that aligns with institutional culture and priorities can lead to outstanding performance and career advancement, enabling individuals to achieve their aspirations and personal goals.

## Background

### Deficit thinking vs growth mindset

For over 30 years, deficit thinking has been a prevalent approach in education. Mainly applied to students of all levels, it focuses on an individual's or group's perceived shortcomings or deficiencies, rather than their strengths or assets. When used in educational or social contexts, deficit thinking can result in unequal treatment and opportunities for specific individuals or groups based on perceived deficits and negative stereotypes rather than their actual abilities and potential [1]. This way of thinking can lead individuals to develop a fixed mindset about their potential, thus limiting their ability to learn. However, if an individual's perspective is changed to focus on their growth as a student rather than their deficiencies, for example, they typically have better academic outcomes. This change in thinking is known as a growth mindset and has been a dominant paradigm in education and psychology for over a decade [2].

The concept of a growth mindset emphasizes how one's perspective influences one's response to criticism, challenges, effort, obstacles, failure, and accomplishments [3]. While the precise effect of growth mindset interventions on academic performance is still under investigation, it is widely accepted that embracing a growth mindset leads to favorable outcomes and achievements. This approach is gaining popularity in mentoring marginalized and underrepresented students, with some research indicating positive results [2, 4–6].

Studies that have explored the application of growth mindset theory to education and faculty development in health and clinical disciplines found beneficial effects on the participants [7–9]. Several studies (reviewed in [10]) have concluded that academics with a growth mindset empower their career success by, among other things, participating in professional development opportunities and reflecting on the outcomes of their activities and adapting their practices (e.g., recognizing and empowering students’ growth mindset potential). However, the impact and potential benefits of applying growth mindset theory on near-professional (e.g., graduate students, health care trainees, postdocs and interns) and early career Science, Technology, Engineering, and Math (STEM) professionals has gained little attention indicating a lack of structured institutional programs from which to draw data [7–9].

Here, we offer a guide to help STEM professionals take charge of their career development through self-guided and peer-supported methods. Using growth mindset theory as a foundation, we present an approach to navigating the early stages of a tenure-track career, including effectively managing multiple responsibilities in teaching, research/scholarship, service, and their intersections. Intended as a practical guide for early-career STEM faculty, this approach is applicable across all institutional classifications and can aid in achieving career success and satisfaction. This guide can also serve as a complement to institutional efforts to foster growth mindset among faculty.

### Growth mindset applied to faculty career development

The process of becoming a STEM faculty member is rigorous and time intensive. For example, in the life sciences and biomedical sciences it requires approximately five years of graduate school and two to five years of postdoctoral experience. During this time, the primary focus is on research, the development of laboratory skills, presentation skills, scientific writing, and foundational knowledge. However, obtaining a faculty position is not the end of the learning process and numerous academic institutions offer support for the professional development of their faculty and staff. Maximizing the benefits of these opportunities requires a keen awareness of the value of ongoing learning, a proactive approach to professional development, and the ability to capitalize on the resources available. By adopting these strategies, aspiring STEM faculty members can enhance their knowledge and skills, positioning themselves for long-term career success.

**Fig. 1:**
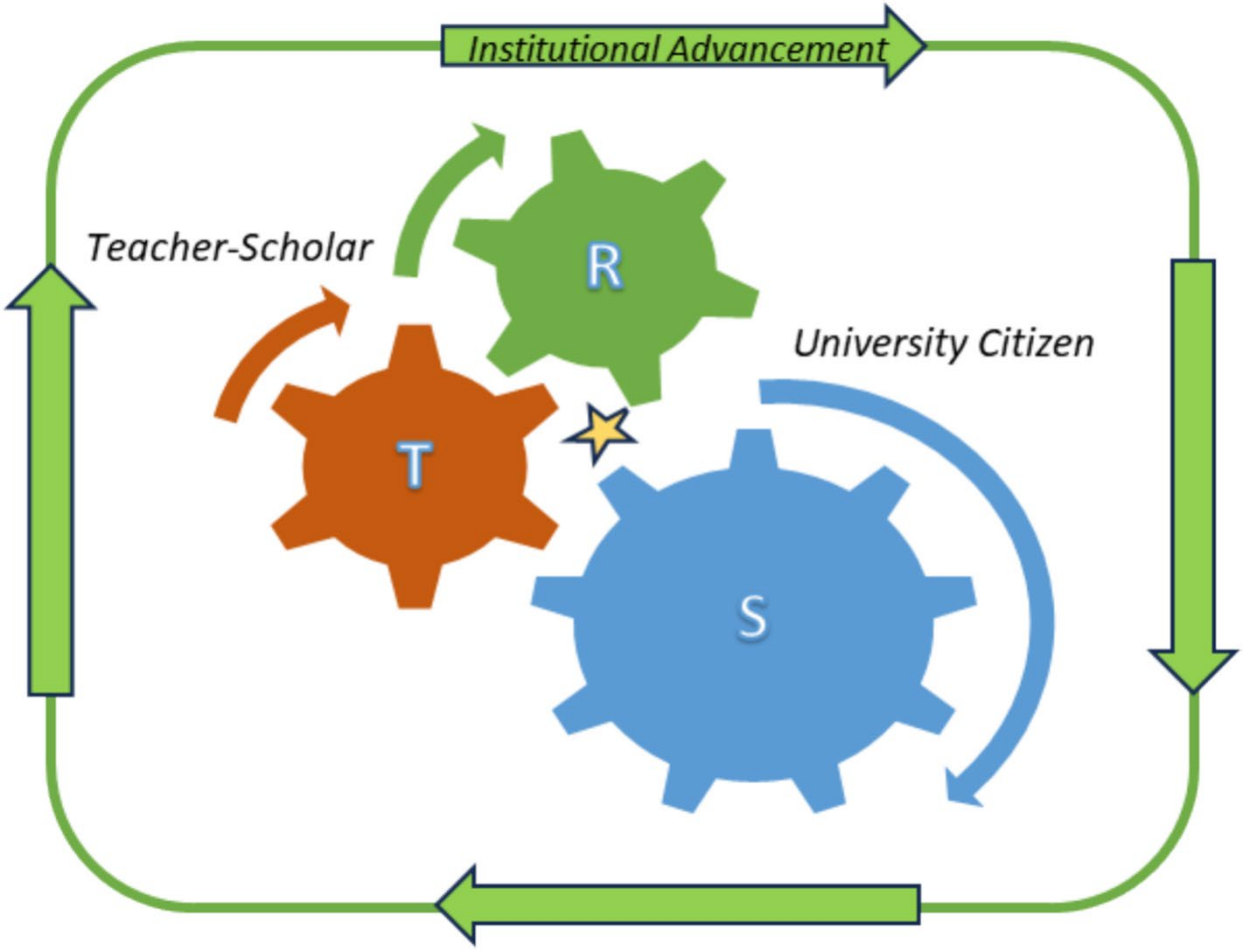
Leveraging service is an integral component of professional growth. The teacher-scholar model advocates for the integration of research (R) and teaching (T) as a creative endeavor to advance student learning and scientific discovery, with benefits for student and faculty. Strategically positioning service (S) as a pivotal gear that synchronously engages with teaching and research (gold star), transforms faculty into valuable university citizens that simultaneously promote student academic success and critical institutional priorities while advancing their own career objectives

While achieving tenure and advancing in rank are common goals for new faculty members, academia offers broader opportunities beyond intellectual pursuits. Institutions often support faculty members in acquiring new skills and taking on varied roles that serve both the institution's goals and the individual's career ambitions. Faculty members aiming for tenure are expected to excel in research or scholarship, teaching and advising, and to a lesser extent service, with the emphasis varying by the institution's focus. While new hires may initially concentrate on the area most aligned with their institution's identity, such as teaching-intensive, research-intensive, or research/teaching achieving, they should not ignore service. New hires also need to recognize that it is not uncommon for faculty of color to carry a heavier service burden than their colleagues [11]. Excessive service commitments will result in workload imbalance that could imperil research progress and teaching effectiveness. Here, we advocate for a growth mindset approach in which service functions as a strategic tool for professional growth and career advancement (Fig. 1). By aligning service commitments with long-term career goals, early career faculty can ensure that their service contributions enhance their research visibility, teaching expertise, and institutional impact rather than becoming an unsustainable burden.

### Growth mindset applied to academic culture

External factors often beyond one's control can significantly impact their career path, especially in academia. The culture within the academic institution, shaped by the leadership of the institution and the actions of the Academic Home Unit (AHU), which includes the department, program, or school, plays a crucial role. It may seem prudent for new faculty to focus solely on research or teaching, temporarily ignoring broader institutional dynamics. However, this approach may result in missed opportunities and a lack of influence on decisions and the cultural evolution within their AHU [12]. By acknowledging the impact of new members influence on workplace culture can empower individuals to contribute to change, enhancing their career progression by aligning with a dynamic academic environment. Being "lucky" in one's career often means being prepared for opportunities when they arise. This preparedness aligns with a growth mindset.

In the next section, we will introduce a growth mindset framework tailored for early career faculty and provide strategies to navigate and capitalize on opportunities across various aspects of their academic roles (Table 1).

**Table 1:** Growth mindset principles and their application at early career situations

Growth Mindset Principles^1^	Situational Opportunity	Rule for Application
1. **Try new strategies**	Assess culture from job interview visit and beyond Benefit from faculty development activities	Contrast expectations with observations; be prepared to go off script; follow-up on pending issues and stay informed Selection and participation guided by workload plan
2. **Meet adversity without fear**	Be a culture change agent	Leverage new faculty status; question more than challenge; constructive conflict engagement
3. **Achievement and success results from efforts**	Learn existing culture and ways to influence change Strategic selection of service committees	Identify cultural components that hinder faculty growth; identify allies for change Contribute to, and benefit from, new initiatives and resource allocation
4. **Mistakes are part of the process**	Redesign an unpopular course	Evaluate student and peer feedback and apply new teaching and learning paradigms
5. **Failures as opportunities to learn, grow and improve**	Unfavorable comments from grant reviewers	Seek interpretation and feedback from trusted colleagues and mentors
6. **Talent and intellect can be grown and developed**	Purposeful utilization of institutional sources of assistance and support	Build skills development and desired outcomes into workload plan
7. **Step out of comfort zone**	Act as an opinion leader Mentor up	Communicate problems and offer solutions or volunteer to seek them Discuss impediments to progress with the AHU chair
8. **Inspired by the success of others**	Participation in thematic cohort activities and in communities of practice Institutional awards	Seek not just to learn but to contribute Serves as a barometer for mutual respect

^1^ Modified from [8]

## Learn, understand, and contribute to academic home unit culture

### Learning culture before and during the job interview visit

A recent COACHE survey highlighted faculty concerns primarily focusing on the culture within academic institutions [13]. Despite not having played a role in shaping the existing culture at their time of hire, new faculty play a crucial role in its evolution. As noted by Gunsalus et al. [14], changing a culture is challenging due to its implicit nature, often summed up as "the way things are." AHU culture can align with the overarching institutional culture or diverge as a distinct subculture or counterculture. Understanding this culture, and the broader academic environment, requires thorough preparation before and engagement during the job interview visit, recognizing its impact on individual career paths as significantly as support from colleagues and leadership. The complexity of organizational culture in academia is underscored by its numerous influencing factors, spanning tangible elements like traditions and intangible aspects such as behaviors prompted by policies [12, 15]. This cultural fabric, akin to the corporate world, represents a significant challenge for institutions aiming for agility and openness to embrace change, highlighting the critical role of the human dimension in academic settings [16]. While academia is often criticized for resisting change, it must cultivate agility and adaptability to navigate its challenges. Recent years have presented academia with many trials, including the COVID-19 pandemic, demographic shifts affecting enrollment and revenue, financial market fluctuations, increased reliance on adjunct faculty, debates over freedom of speech, ideological conflicts, and political pressures. These seismic events catalyze profound cultural shifts, separating institutions that adapt from those that falter, with some even facing financial exigency or closure [17].

Regardless of their status, every individual within an institution holds the power to influence their culture significantly. But prospective faculty members are pivotal in assessing an institution's response to crises and culture. While institutions may not provide explicit documents detailing their units’ culture, candidates can glean valuable insights through preparedness and astute questioning. Observations of physical spaces, particularly public displays that celebrate the unit’s values and accomplishments of its members, interactions among faculty and staff, and the compositional diversity of the AHU offer further clues. In summary, information gained during the job interview visit allows the candidate to identify institutional opportunities, leverage a growth mindset to adapt to institutional realities**,** and better position themselves to take advantage of resources, rather than being constrained by existing limitations.

### Gaining culture understanding during early years and finding a place in it

The inception of a new position marks the transition of the former candidate from an external observer to an integral part of the AHU community, vested with the opportunity and responsibility to shape its culture. Early engagement necessitates astute observation and a commitment to ongoing learning, as the insider perspective unveils nuances of the culture previously concealed to outsiders. Key among these observations is the dynamic interplay among AHU faculty members and their interactions with students, staff, and academic leaders, all offering invaluable insights into the prevailing ethos. Equally significant is discerning their attitudes toward teaching, research, service, and institutional priorities, reflecting their dedication to these core responsibilities, and illuminating their perception of their roles in advancing collective objectives.

Moreover, delving into recent and historical trends can yield additional illumination. For instance, tracking the prevalence of institutional awards among AHU members extends beyond mere acknowledgment of their professional accomplishments; it serves as a barometer of mutual respect and camaraderie within the community. Recognition through nominations for such awards often originates from within the AHU, underscoring the depth of its internal cohesion. Conversely, a dearth of recognized achievements may signal a deficiency in collaboration, solidarity, and engagement, potentially leading to marginalization within the institution's broader discourse and further eroding the AHU's sense of identity and value. In this regard, every member plays a pivotal role in shaping the unit's culture. A collaborative environment, where each member contributes meaningfully, fosters resourcefulness, productivity, and sustainability, thereby nurturing the career progression of all involved. Faculty meetings serve as particularly poignant reflections of AHU culture, offering insights into its priorities, challenges, and the level of faculty engagement. These gatherings also provide unfiltered glimpses into interpersonal dynamics, communication styles, and the prevailing norms of conduct.

It is imperative, however, to exercise caution in interpreting these interactions. A newcomer may initially perceive vigorous debates and assertive exchanges as discordant or confrontational, unaware that such exchanges are integral to the community's collaborative ethos. Analogous to competitive athletes displaying intensity during a game only to reconcile afterward, these interactions may represent spirited exchanges of ideas rather than personal attacks. Evaluating the outcome of such exchanges, including the emergence of constructive ideas and collaborative follow-up actions, offers crucial insights into the health and functionality of the culture. Furthermore, it is essential to recognize that while academic freedom and the right to free expression are fundamental, they must not be conflated with personal attacks or disruptive behavior [18]. Vigilance against repeated instances of instigation and proactive measures to address them is vital for maintaining a constructive environment. Importantly, new AHU members are encouraged to embody the behaviors they wish to see in others while respecting boundaries. By identifying and aligning with colleagues committed to fostering a positive culture, they contribute to the collective endeavor of cultivating a thriving academic community (Table 1, principles 3, 7).

In summary, understanding and actively shaping institutional culture is not peripheral but central to adopting and implementing a growth mindset for early-career STEM faculty. Institutional culture establishes the conditions in which faculty either flourish or stagnate. By actively participating in shaping their institutional culture, as introduced in the next section, faculty leverage growth mindset principles to align individual professional growth with broader institutional transformation, creating environments that explicitly support continued learning, risk-taking, and innovation rather than compliance-driven, prescriptive metrics of success.

### Why (and How to) contribute to solutions and culture change

Once part of the AHU, the newly hired faculty can shape their unit's culture. While it's common advice to focus on promotion and tenure, new faculty should not completely ignore institutional "politics" and should stay attuned to decisions that may adversely impact career progression. A working AHU appoints members, through committee assignments, to track developments in different institutional divisions and report back to the unit for discussion and feedback. This information can be discussed in regular faculty meetings to provide feedback and recommendations to institutional divisions through the appointed member. By staying informed and letting their voice be heard, the new faculty members can shape institutional decisions that affect unit culture and, by extension, their career (Table 1, principle 1).

Academic leaders have a vested interest in the growth and development of their new faculty and are eager to remove any obstacles that may hinder their progress. Early career faculty members should take advantage of opportunities, formal and informal, to interact with academic leadership and express their opinions on topics that directly affect them. It's important to be prepared to take ownership and offer workable solutions when doing so (Table 1, principles 6 and 7). However, new faculty members should not be expected to solve problems on their own. It is crucial to recognize the time commitment involved and prioritize tasks before taking on such responsibilities. Experienced individuals and those in administrative roles should take the lead. Instead, new faculty members can contribute as opinion leaders or by offering well-informed advice based on their experiences, thus working collaboratively toward achieving better performance. Overcoming the temptation of stepping back to let others fix the problem is a necessary step for improving the landscape. In the words of Mica Estrada—“*Weeds grow in places where there’s nothing healthy growing*” [19].

Individuals are encouraged to voice their concerns about broader problems that affect the community during annual reviews or program reviews. When sharing concerns, it is important to propose a workable solution or a different perspective on the root cause of the problem when sharing concerns. Rational and carefully thought-out recommendations delivered in a cooperative, collegial tone that accentuates a better future are more likely to resonate with leaders than aggressive attacks. The benefits of speaking outweigh the risks of remaining silent, particularly for individuals who belong to underrepresented groups and may not feel like they are part of the community, and their voices are not well represented (Table 1, principle 7). To quote civil rights icon and long-term Congressman John Lewis, “*Never, ever be afraid to make some noise and get in good trouble, necessary trouble*.”

## Institutional structure and culture

### Institutional structure

**Fig. 2:**
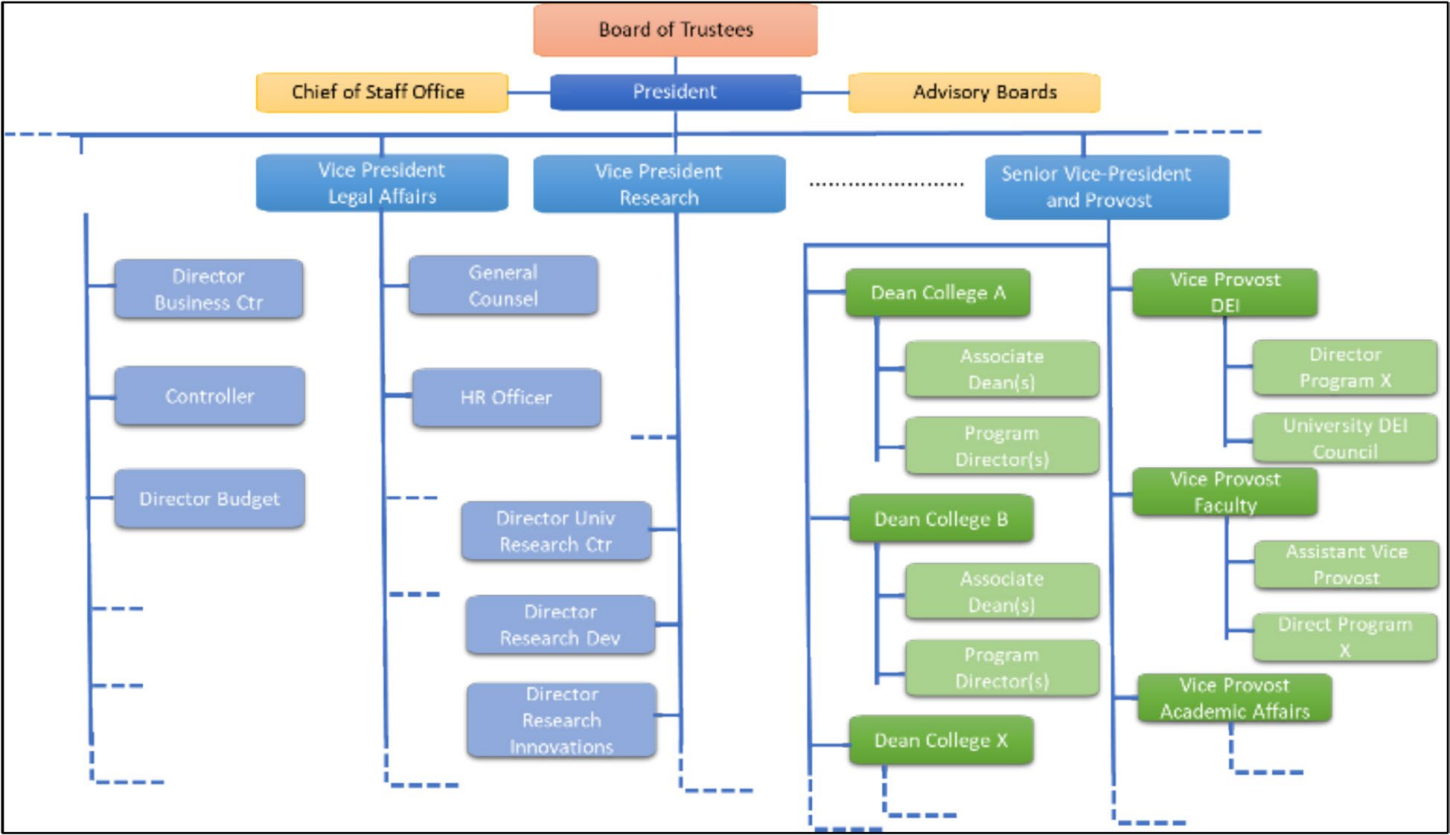
Simplified example of an academic organization chart. The example includes only some major divisions that play significant roles in the institution's governance and operation. Positions that report to the president are shown in blue and the ones that report to the senior vice-president/provost are shown in green. Broken and dotted lines indicate additional divisions and sub-divisions (not shown)

Organizational charts depict the hierarchical reporting lines and connections between senior leaders and administrative and academic units in an institution (Fig. 2). These charts are frequently updated to reflect leadership transitions and structural modifications and are considered dynamic documents. It serves the new faculty member to comprehend the institutional structure and the responsibilities and resources of decision-makers to advance their career. Realignments within an institution reflect strategic priorities and resource allocation, so it is necessary to pay close attention to any changes. New faculty members should make their voices heard during restructuring.

### Institutional culture

The public statements and internal communications of institutional leaders offer a window into an organization's culture and values. It is crucial to pay attention to the issues that an institution prioritizes in its communications, as well as the tone and angle of its position. National issues that impact academia reflect an institution's cultural soul and values, which are reflected in its history, mission, vision, strategic priorities, fundraising themes, and action plans. When new faculty members are seen as valuable contributors to these declarations, they are more likely to achieve career success. Understanding how the institution's processes generate sustainable change and the extent to which faculty members contribute to these processes is essential.

It is important to approach institutional issues with care and balance, avoiding both overreaction and underreaction. Is the AHU a microcosm of the institution's struggles, or an oasis that thrives despite surrounding chaos? Understanding the situation will dictate with whom and how the new faculty member should engage and what resources to tap into (see next section). By recognizing their role in advancing positive change and taking a measured approach to institutional challenges, faculty members contribute to creating a vibrant and successful academic community.

Faculty who find themselves in unsupportive or toxic environments face distinct challenges in successfully applying growth mindset principles. In these cases, employing a growth mindset involves identifying strategies that minimize the negative effects of these environments and maintaining professional momentum. This might include seeking mentorship and community beyond the immediate academic unit, leveraging external professional networks, proactively engaging institutional resources designed to assist faculty (such as external faculty development centers, employee assistance programs, or ombudspersons), and critically reframing negative experiences and setbacks as opportunities to develop resilience and adaptive skills*.*

### Sources of assistance and support

Numerous academic institutions provide faculty development events and resources. While most focus on enhancing teaching and advancing institutional goals for student achievement, others address research and leadership skills (Table 1, principles 1 and 6). These activities not only promote skills development but also provide opportunities to establish connections with colleagues who share similar interests (Table 1, principle 8). To ensure the optimal benefit of these opportunities, it is important to choose them carefully, as the array of options can be overwhelming. Ideally, they should be incorporated into a broader plan developed with a direct supervisor (e.g., department chair, program director, or associate dean, herein AHU chair) and mentors (Sect. 4). Participants in programs that include colleagues with similar interests typically feel valued by their institutions and report lower odds of burnout [20]. Awareness of institutional resources and opportunities signifies an institutional commitment to the success of faculty careers. However, academics are often confronted with challenges when seeking assistance with specific issues, whether involving a student, colleague, or supervisor or when wanting to capitalize on an opportunity for which they lack the know-how. The primary concern is not the availability of support but rather the lack of familiarity with the institutional structure and where to locate appropriate resources. In Table 2, we provide examples of typical institutional resources, their locations, and external resources. A secondary concern is the timing of accessing these resources; proactively, as laid out in a career plan, or reactively, after a concern has been noted about the individual’s performance. For example, an early-career faculty with little teaching experience may face poor student performance and negative course evaluations if they had not sought assistance in preparation for their course. Resources and mentorship from the Center for Teaching and Learning help implement strategies that, over time, result in improved student learning and course evaluations. Furthermore, studies have shown that a teacher’s growth mindset is positively associated with the development of student growth mindset [21], potentially leading to better student outcomes.

**Table 2:** Examples of internal and external resources for early career faculty*

Entity	Description	Notes
Institutional Resources		
Division of Faculty Affairs	Procedures and policies impacting faculty; resources for professional development	Some professional development resources may be overseen by other units
Center for Teaching & Learning	Support on matters of instructional design, pedagogy, and learning management systems	May include faculty-led groups, communities of practice
Office of Research/Sponsored Programs	Internal and external grants & awards; support for proposal submissions; pre- and post-award services	May offer training (e.g., grant writing, budgeting)
Human Resources (HR)	Benefits, Employee Wellness Programs	Health and financial well-being
Labor Employee Relations	Mediate situations between institution and employee or representative union	Issues related to collective bargaining agreement; may be subdivision of HR;
Employee Assistance Programs	Confidential consultations on personal and relational issues	Outsourced to emphasize confidentiality
Faculty Senate	Faculty rights and policies governing curriculum; shared governance	Elected and representative body
Ombudsperson	Assists faculty with resolution of conflicts and complaints	Similar but less formal roles served by pracademics [22, 23]
Office of Engagement	Fosters partnerships with community, private, public, and non-profit organizations	Funding opportunities for faculty may be available
Office of Service Learning	Resources and support for implementing community-engaged courses, experiential learning	Some funding opportunities may be available
Office of Innovation	Assistance with intellectual property, technology transfer and entrepreneurship	May provide training and financial resources
Foundation	Philanthropy and capital campaigns	May assist with private financing for research and pedagogy
External Resources		
Chronicle of Higher Education	https://www.chronicle.com/career-resources/	General information & professional development articles; Institutional subscription
Inside Higher Ed	https://www.insidehighered.com/opinion/career-advice	General information & professional development articles; some content free
Association of Public and Land Grant Universities (APLU)	https://www.aplu.org/	Professional and informational resources
National Center for Faculty Development and Diversity (NCFDD)	https://www.ncfdd.org/home	Professional development programs; individual and institutional memberships
Howard Hughes Medical Institute (HHMI)	https://www.hhmi.org/	Support for advancing research and science education
American Society for Cell Biology (ASCB) ACT	https://www.ascb.org/career-development/accomplishing-career-transitions-act-program/	Professional development training program; other scientific societies offer similar programs
National Center for Principled Leadership & Research Ethics	https://ncpre.csl.illinois.edu/	Resources to assist challenged units

^*^Names and description of institutional resources are examples and vary from institution to institution

## Career strategy and the workload plan

New faculty members can benefit greatly from building a strong relationship with their AHU chair, who oversees their workload and development. Understanding the AHU chair's leadership style and whether they prioritize maintaining the status quo or promoting change can be helpful. It is also important to consider whether the current AHU culture is a result of a former visionary chair's legacy or subsequent chairs who are less invested in the AHU's goals and vision as described in Sect. 2.

### Role of AHU chair and how they got there

AHU chairs are often categorized as mid-level leadership positions situated between academic faculty and administrators. It is a challenging job that few want, given its sometimes hidden complexity and unpredictability [24]. The AHU chair is responsible for advocating and protecting the early career faculty and ensuring their success. However, many are not adequately prepared to take on this significant responsibility, resulting in a limited ability to initiate and sustain meaningful change [25]. Therefore, it is no surprise that the chair position is regarded by many as the most challenging leadership role in academia.

Understanding the duration of a chair's tenure and the circumstances surrounding their appointment can provide valuable insights for early career faculty who are seeking to form a partnership. In research-intensive institutions, chairs are typically hired after conducting a national search based on their research accomplishments, credentials, and ability to lead the AHU toward becoming a research powerhouse. They usually come with a wealth of experience, a clear agenda, and a promise of resources to help execute their vision. The expectation is that this will be a long-term commitment. On the other hand, many schools hire chairs internally, usually from among AHU members. These appointments tend to be for a defined period (typically 3–5 years but can be longer) and passed from one AHU member to another. These “rotating chairs” are experienced AHU members who may be more approachable and open-minded to ideas and unsolicited advice from colleagues. However, they may be less inclined towards radical change unless it is mandated from above.

An AHU chair chosen solely on their teaching or research accomplishments may not always result in the best selection. Good leadership qualities are not always indicative of these accomplishments. Another factor to consider is the relationship between the chair and their direct supervisor, e.g., the dean. If the chair always agrees with the dean's decisions, it could suggest that the dean is autocratic, or the chair is solely focused on maintaining the status quo.

For early career faculty members, building a healthy relationship with the AHU chair is crucial. This will demonstrate commitment to improving the workplace and provide valuable feedback to help the chair grow as a leader. Keep in mind that chairs have many academic and administrative responsibilities, which can sometimes detract from their leadership role. Therefore, it is important to take advantage of opportunities for providing constructive feedback, whether through formal evaluations or informal discussions. **(**Table 1, principle 7).

In summary, to establish a productive relationship with an AHU chair, mutual respect and bidirectional mentoring should be established. This relationship provides a solid foundation for the strategy of career success through deliberate attention to planning the workload.

### Approaching the workload plan

Tenure track academics generally have responsibilities assigned to teaching, research, and service in various percentages of effort. Teaching combined with research normally account for most of the work effort, 80% or more. The smaller percent effort assigned to service, which can encompass service at various institutional levels as well as service to the profession, is interpreted by some as simply being a box that needs to be checked and being of little to no consequence in promotion decisions and in contributing to career success. The 20% or so annual effort commitment to service provides, over time, ample opportunities for career building if approached strategically and with a plan. Sharing the plan with the AHU chair and mentor(s) is one way of holding oneself accountable and thus more likely to execute the plan successfully.

A common advice to early career faculty is to keep service to a minimum. In extreme cases, early career faculty are “protected” from engaging in service as it is viewed as imperiling progress in research at a critical stage of establishing an independent career. When service is allowed, it is often hand-picked (colloquially “voluntold”) or heavily influenced by the AHU chair for an activity or committee that requires little time commitment. This decision comes with the clear benefit of time saving, a rare commodity in academia, but it also comes with the cost of missed opportunities for professional growth and career advancement. Every workload decision should have strategic implications and as such should be made with career goals in mind.

There are two major considerations to be made when deciding on options to fulfill service obligations and advance career goals—skill development and goal congruency. The former refers to service participation that provides space for learning by exposing the individual to principles, practices, and processes in institutional decision making. Gaining skills in these areas positions the individual to better identify opportunities and resource utilization that can advance their career (Fig. 1 and Table 1, principle 3). For example, an individual who is interested in engaging undergraduates in research may want to understand (and influence) how decisions are made regarding designation of resources towards course-based undergraduate research. This may entail participation in the curriculum committee or a curricular task force (Table 3), an opportunity that can additionally provide insight into the students’ perception, preparation, and expectations of undergraduate research, knowledge that can inform strategies for effective recruitment and mentoring of students and, by extension, increased research productivity.

**Table 3:** Aligning service opportunities and institutional priorities with career goals: hypothetical examples

Early Faculty Career Goal	Service Option*	Institutional Goal
Instructional Innovation	Teaching & Learning Fellows	Promote Culture of Academic Engagement
Curricular Quality & Innovation	Implementation Task Force	Prepare Students for Diverse Careers; Create New Major
Parity in Student Outcomes	Inclusive Classroom Community of Practice	Improve First-year Retention and Four-year Graduation Rate
Increase Undergraduate STEM Participation	Curriculum Committee	Promote Effective Classroom Practices and Undergraduate Research
STEM Training & Workforce Development	Graduate Program Committee	Enhance Research & Scholarship
Excellence in Pedagogy	Teaching Awards Committee	Recognition & Celebration of Faculty Accomplishments
Commitment to Student Success	Learning Communities Advisory Group	Student Retention & Academic Success
Biomedical Innovation	Advisory Committee to VP for Research	Improve Health and Wellness
Participatory Action Research	Program for Community Engagement	Serve needs of the Community, State, and Nation
Science Policy	Commission on Higher Education	Partnership with State Leaders and Legislators
Public Health	Liaison Group with Policy Makers	Solve Local and Global Challenges
Academic Leadership & Policy	Faculty Senate	Shared Governance Principle

^*^ Additional service options are available through professional organizations and societies

Goal congruency in the context of early career planning refers to matching personal goals with AHU and institutional priorities. According to goal congruency theory, individuals are more likely to achieve goals that align with their values [26]. Seeking congruency with institutional goals has multiple benefits, namely: (*i*) opportunity to influence decisions that can directly impact the individual’s career trajectory; (*ii)* positioning the individual to maximize access opportunities to programs and resources relevant to their career goals; and (*iii*) increased feelings of belonging [27].

Approaching the workload conversation with the AHU chair having an outline of short and long-term career goals will facilitate identifying service opportunities congruent with those goals. The approach to the conversation should be one of *questioning-up*. To start, the individual should describe their interests, skills, and career goals. Having shared this information, the individual can then ask about possible committee assignments that can support those goals while simultaneously benefiting the AHU. Once a few options are discussed, the individual can then ask what the best outcome for the AHU and the institution would be if they were to serve on a particular committee, considering the knowledge, skills, and experience they can bring to the committee.

### AHU operational boundaries

Finally, one must consider that AHUs have varying degrees of autonomy over their operations, including decisions on reappointment, promotion, tenure, sabbaticals, workload, space allocation, and fund usage. While AHUs play a significant role in hiring and retention, decisions are often made based on outdated trends. Early-career faculty can contribute fresh perspectives to AHU governance, influence their workplace, and impact the unit's reputation.

## Closing thoughts

Climbing the ladder of career success in academia should not be limited to “check box” accomplishments for achieving tenure or the rank of full professor. There are many opportunities for growth and contribution outside the traditional path of tenure and rank promotion that will enrich the journey and amplify the impact a career faculty can have on their profession. Although it is impossible to plan for every potential opportunity, maintaining a growth mindset helps the individual expand their love for learning, transform challenges into opportunities, and seek improvements in multiple facets of their career. Early career faculty who actively model and communicate a growth mindset in their research, teaching, and mentorship interactions can cultivate resilience in themselves and their students. By integrating reflective practices, such as discussing setbacks and iterative problem-solving in research and coursework, early career faculty can create a culture of persistence that benefits both their own professional growth and their students' academic trajectories. By aligning their goals with those of their institution and professional organization(s), the early career faculty can work towards achieving both their objectives and those of the organizations they serve. Recognition and celebration of the achievements accomplished by the individual will only help elevate their career, self-esteem, and sense of belonging.

## Data Availability

Not applicable.

## Abbreviations

STEM: Science, Technology, Engineering, and Mathematics
AHU: Academic Home Unit
COACHE: Collaborative on Academic Careers in Higher Education
ASCB: American Society for Cell Biology

## References

[1] Patton Davis L, Museus S. What is deficit thinking? An analysis of conceptualizations of deficit thinking and implications for scholarly research. Currents. 2019;1(1):117–30.

[2] Dweck CS. Mindset: The new psychology of success. Random House; 2006.

[3] Ku YR, Stager C. Rethinking the multidimensionality of growth mindset amid the COVID-19 pandemic: a systematic review and framework proposal. Front Psychol. 2022;13:572220. 35846666 10.3389/fpsyg.2022.572220 PMC9284032

[4] Sisk VF, Burgoyne AP, Sun J, Butler JL, Macnamara BN. To what extent and under which circumstances are growth mind-sets important to academic achievement? Two meta-analyses. Psychol Sci. 2018;29(4):549–71. 29505339 10.1177/0956797617739704

[5] Harper SR. In: *New Directions for Institutional Research.* Wiley Periodicals, Inc.; 2010: 63–74.

[6] Yeager DS, Dweck CS. Mindsets that promote resilience: when students believe that personal characteristics can be developed. Educ Psychol. 2012;47(4):302–14.

[7] Wolcott MD, McLaughlin JE, Hann A, Miklavec A, Beck Dallaghan GL, Rhoney DH, et al. A review to characterise and map the growth mindset theory in health professions education. Med Educ. 2021;55(4):430–40. 32955728 10.1111/medu.14381

[8] Carlson ER, Tannyhill RJ. The growth mindset: a contextualization of faculty development. J Oral Maxillofac Surg. 2020;78(1):7–9. 31525329 10.1016/j.joms.2019.07.020

[9] Shapiro N, Dembitzer A. Faculty development and the growth mindset. Med Educ. 2019;53(10):958–60. 31509287 10.1111/medu.13948

[10] Sousa BJ, Clark AM. Growth mindsets in academics and academia: a review of influence and interventions. J High Educ Policy Manag. 2024;47(1):38–56.

[11] Griffin KA. Redoubling Our Efforts: How Insitutions Can Affect Faculty Diversity. In: Espinosa LL, Turk JM, Taylor M, Chessman HH, editors. Race and Ethnicity in Higher Education: A Status Report. American Council on Education; 2019. pp. 273–279. www.equityinhighered.org.

[12] Tierney WG. Organizational culture in higher education: defining the essentials. J High Educ. 1988;59(1):2–21.

[13] Mathews K, Benson T. COACHE Data: Faculty Says Culture is the Top Thing to Address to Improve the Workplace. 2023. https://coache.gse.harvard.edu/blog/coache-data-faculty-says-culture-top-thing-address-improve-workplace. Accessed 01 Apr 2023.

[14] Gunsalus CK, Luckman EA, Burbules NC, Easter RA. How to change and unhealthy department culture. 2019. https://www.insidehighered.com/advice/2019/03/14/recommendations-improving-unhealthy-department-culture-opinion. Accessed 24 Apr 2023.

[15] Schein EH. Organizational culture and leadership. 1st ed. San Francisco: Jossey-Bass Publishers; 1985.

[16] Jurisic N, Lurie M, Risch P, Salo O. Doing vs being: Practical lessons on building an agile culture. 2020. https://www.mckinsey.com/capabilities/people-and-organizational-performance/our-insights/ng-vs-being-practical-lessons-on-building-an-agile-culture. Accessed 15 May 2023.

[17] Nietzel MT. More Colleges, Universities Announce Budget Cuts Amid Financial Woes. In: *Forbes.* 2023. https://www.forbes.com/sites/michaeltnietzel/2023/09/30/more-colleges-universities-announce-budget-cuts-amid-financial-woes/?sh=63604bf15507. Accessed 01 Apr 2024.

[18] Sutton RI. The No Asshole Rule: Building a Civilized Workplace and Surviving One That Isn't. Balance; 2010.

[19] Estrada M. How kindness matters for diversifying stemm fields. 2023. https://ids.wisc.edu/2023/10/05/diversity-science-podcast-mica-estrada-on-how-kindness-matters-for-diversifying-stemm-fields/. Accessed 5 Oct 2023.

[20] Giess CS, Ip IK, Gupte A, Dudley JC, Healey MJ, Boland GW, et al. Self-reported burnout: comparison of radiologists to nonradiologist peers at a large academic medical center. Acad Radiol. 2022;29(2):277–83. 33172814 10.1016/j.acra.2020.10.013

[21] Mesler RM, Corbin CM, Martin BH. Teacher mindset is associated with development of students’ growth mindset. J Appl Dev Psychol. 2021;76:101299.

[22] Hollweck T, Netolicky DM, Campbell P. Defining and exploring pracademia: identity, community, and engagement. J Prof Cap Commun. 2022;7(1):6–25.

[23] Volpe MR, Chandler D. Resolving and managing conflicts in academic communities: The emerging role of the “pracademic.” Negotiation J. 2001;17(3):245–55.

[24] Zahneis M. The faculty job (almost) no one wants. In: *The Chronicle of Higher Education.* 2022. https://www.chronicle.com/article/the-faculty-job-almost-no-one-wants. Accessed 4 Jan 2024.

[25] Eddy PL, Garza Mitchell R, Amey MJ. Leading from the middle. In: *The Chronicle of Higher Education.* 2016. https://www.chronicle.com/article/leading-from-the-middle/. Accessed 13 Dec 2023.

[26] Sansone C, Sachau DA, Weir C. Effects of instruction on intrinsic interest: the importance of context. J Pers Soc Psychol. 1989;57(5):819–29. 2810027 10.1037//0022-3514.57.5.819

[27] Estrada M, Eroy-Reveles A, Matsui J. The influence of affirming kindness and community on broadening participation in STEM career pathways. Soc Issues Policy Rev. 2018;12(1):258–97. 29657577 10.1111/sipr.12046 PMC5898245

